# Supplementary material for: Variations in Children’s screen time behaviors by weight status among a mostly disadvantaged population
Source: PLoS One. 2026 Apr 17;21(4):e0346235. doi: 10.1371/journal.pone.0346235 (PMC13089690; doi:10.1371/journal.pone.0346235)
Supplement: Attachment — Submitted filename: Response to reviewers.docx [file pone.0346235.s002.docx]

When submitting your revision, we need you to address these additional requirements.

1. Please ensure that your manuscript meets PLOS ONE's style requirements, including those for file naming. The PLOS ONE style templates can be found at <https://journals.plos.org/plosone/s/file?id=wjVg/PLOSOne_formatting_sample_main_body.pdf> and <https://journals.plos.org/plosone/s/file?id=ba62/PLOSOne_formatting_sample_title_authors_affiliations.pdf>

Response: All requirements are met. Thank you.

2. Your ethics statement should only appear in the Methods section of your manuscript. If your ethics statement is written in any section besides the Methods, please delete it from any other section.

Response: Deleted as advised.

3. & 4. Regarding the data availability statement.

Response: We have revised and resubmitted the Data Availability Statement to clearly indicate that the data are available only upon request and contingent on an IRB-approved data-sharing agreement.

5. Regarding funders’ roles.

Response: The funders had no role in study design, data collection and analysis, decision to publish, or preparation of the manuscript.

6. If the reviewer comments include a recommendation to cite specific previously published works, please review and evaluate these publications to determine whether they are relevant and should be cited. There is no requirement to cite these works unless the editor has indicated otherwise.

Response: Thank you. Reviewers did not recommend citations.

**Comments to the Author**

1. Is the manuscript technically sound, and do the data support the conclusions?
The manuscript must describe a technically sound piece of scientific research with data that supports the conclusions. Experiments must have been conducted rigorously, with appropriate controls, replication, and sample sizes. The conclusions must be drawn appropriately based on the data presented.

Reviewer #1: Yes

Reviewer #2: Yes

Reviewer #3: Yes

2. Has the statistical analysis been performed appropriately and rigorously?

Reviewer #1: Yes

Reviewer #2: Yes

Reviewer #3: No

Author response: We have carefully revised the data analysis.

3. Have the authors made all data underlying the findings in their manuscript fully available?
The [PLOS Data policy](https://nam04.safelinks.protection.outlook.com/?url=http%3A%2F%2Ftrack.editorialmanager.com%2FCL0%2Fhttp%3A%252F%252Fwww.plosone.org%252Fstatic%252Fpolicies.action%2523sharing%2F1%2F010f019bb4ec29a9-d17d75d9-559f-4581-bd85-1b311b696ec5-000000%2FlAMBR9zeE8GSZDRoOn1B1Iyo69iuZJb6uwGiV8dxksw%3D245&data=05%7C02%7Cdson2%40lsu.edu%7C22130f7fecb1422d64fc08de52af6979%7C2d4dad3f50ae47d983a09ae2b1f466f8%7C0%7C0%7C639039110497265862%7CUnknown%7CTWFpbGZsb3d8eyJFbXB0eU1hcGkiOnRydWUsIlYiOiIwLjAuMDAwMCIsIlAiOiJXaW4zMiIsIkFOIjoiTWFpbCIsIldUIjoyfQ%3D%3D%7C0%7C%7C%7C&sdata=qLdWDjez1WuKxK4dbPGSQc2mprU%2BEMC8v%2FdQ2mSHwQY%3D&reserved=0) requires authors to make all data underlying the findings described in their manuscript fully available without restriction, with rare exception (please refer to the Data Availability Statement in the manuscript PDF file). The data should be provided as part of the manuscript or its supporting information, or deposited to a public repository. For example, in addition to summary statistics, the data points behind means, medians and variance measures should be available. If there are restrictions on publicly sharing data—e.g. participant privacy or use of data from a third party—those must be specified.

Reviewer #1: No

Reviewer #2: Yes

Reviewer #3: Yes

Author response: We have revised and resubmitted the Data Availability Statement to clearly indicate that the data are available only upon request and contingent on an IRB-approved data-sharing agreement.

4. Is the manuscript presented in an intelligible fashion and written in standard English?
PLOS ONE does not copyedit accepted manuscripts, so the language in submitted articles must be clear, correct, and unambiguous. Any typographical or grammatical errors should be corrected at revision, so please note any specific errors here.

Reviewer #1: Yes

Reviewer #2: Yes

Reviewer #3: Yes

5. Review Comments to the Author
Please use the space provided to explain your answers to the questions above. You may also include additional comments for the author, including concerns about dual publication, research ethics, or publication ethics. (Please upload your review as an attachment if it exceeds 20,000 characters)

**Reviewer #1**: This manuscript examines differences in screen-time behaviors by weight status in a socioeconomically disadvantaged pediatric population. The topic is relevant, the sample size is adequate, and the use of a multidimensional screen-time instrument is a strength.

The analyses are generally appropriate; however, several issues require clarification or revision before the manuscript can be considered for publication.

Response: Thank you for your insightful review of our paper and for recognizing its merit. We have carefully addressed your comments and provide below our responses to your comments point by point.

1. **First, causal or directional language should be revised throughout the manuscript, as the cross-sectional design only allows for assessment of associations**

Response: We reviewed the manuscript and revised causal or directional wording to ensure that all interpretations appropriately reflect associations, not causations. Specifically, we replaced phrases implying risk, attribution, or contribution (e.g., “puts children at elevated risk,” “attributable to,” “contribute to”) with association-based language (e.g., “was associated with,” “were more likely to,” “differences were most pronounced”). These revisions were made in the Abstract, Introduction, Discussion, and Conclusions to improve clarity and align the language with the study design.

2. **Second, the Data Availability Statement does not clearly comply with PLOS ONE policy. The manuscript alternately states that data are available within the manuscript and that datasets require an IRB-approved data-sharing agreement. This inconsistency must be resolved.**

Response: Thank you for pointing out the inconsistency regarding our data-sharing statement. We have revised and resubmitted the Data Availability Statement to clearly indicate that the data are available only upon request and contingent on an IRB-approved data-sharing agreement.

3. **Third, although multiple statistically significant findings are reported, many effect sizes are small. The authors should more clearly discuss the practical or public-health relevance of these differences.**

Response: We appreciate this helpful observation and agree that several statistically significant differences were associated with small effect sizes. In the revised manuscript, we have expanded the Discussion to acknowledge the modest magnitude of the observed effect sizes, and the practical and public health relevance should be interpreted with caution. We further emphasize that, while individual differences in daily screen time were relatively small to moderate, these differences may still be meaningful at the population level. We further explain that an average difference of approximately 60 minutes per day for weekday passive screen time represents a potentially relevant behavioral target for intervention.

4. **Fourth, parts of the discussion and conclusions place emphasis on weekend screen time, despite results indicating that the largest weight-status differences occur for weekday passive screen time. The interpretation and recommendations should be more closely aligned with the results.**

Response: We agree that our primary findings indicate that the largest weight-status differences in screen time occurred for passive screen use on weekdays. Therefore, we have clarified the Discussion and Conclusions that weekday passive screen time represents the most pronounced disparity by weight status and emphasize this pattern is the primary target for intervention, particularly during after-school hours.

The discussion around the weekend screen time is retained as it relates to the *structured day hypothesis* (SDH), which is an important theoretical underpinning for child health and health behavior research. This discussion has been downplayed to be consistent with the focus.

5. **Finally, minor editorial issues (typographical errors and repetitive phrasing) should be corrected.**

Response: We went through the manuscript and corrected typographical errors, clarified wording, and reduced repetitive phrasing throughout the text. For example, we corrected typographical errors (e.g., “engaged” to “aged,” “spent” to “spend”), duplicated words such as “Nevertheless, Nevertheless,” or unclear phrasing to improve clarity and readability.

Overall, the study addresses an important question, but the issues noted above should be addressed to improve clarity, transparency, and interpretability.

**Reviewer #2**: This manuscript as a novel approach examines multidimensional screen time behaviors among children with overweight/obesity (OWOB) compared to their normal-weight peers in a socioeconomically disadvantaged population. So, congratulations to the research team for taking a good initiative study and overall, the study addresses an important and relevant public health issue making meaningful contribution to the literature by moving beyond total screen time to examine engagement type, posture, and weekday versus weekend patterns.

With these, I have few broad queries and suggestions as part of preliminary screening and I hope the research team will address it adequately.

Response: Thank you for your insightful review of our paper. Changes have been made in the manuscript, and we have carefully addressed your comments and provide below our responses to your comments point by point.

1. **Please clarify throughout the discussion and conclusion that the findings represent associations only, given the cross-sectional study design, and avoid any language that may imply causality.**

Response: Thank you. We have revised causal or directional wording to ensure that all interpretations appropriately reflect associations, not causations. Specifically, we replaced phrases implying risk, attribution, or contribution (e.g., “puts children at elevated risk,” “attributable to,” “contribute to”) with association-based language (e.g., “was associated with,” “were more likely to,” “differences were most pronounced”). These revisions were made in the Abstract, Introduction, Discussion, and Conclusions to improve clarity and align the language with the study design. Additionally, we revised the Conclusion to explicitly clarify that the findings reflect associations only and do not imply causation, consistent with the cross-sectional study design.

2. **Write a few lines about the potential impact of parent-reported screen time and anthropometric data, particularly the possibility of differential reporting, and how this may influence the results.**

Response: Thank you for this important suggestion regarding the issue of precise measurement. We have added a paragraph to the Discussion acknowledging the potential impact of parent-reported screen time and anthropometric data, including the possibility of differential reporting by child weight status. We note that recall errors and misreporting height and weight of their child may have influenced the observed associations. In addition, we discuss the value of future studies incorporating objective measures to strengthen measurement precision.

3. **In the findings section, clarify the interpretation of the non-significant moderation effects by demographic variables, including whether limited variability within the sample or statistical power may have contributed to these findings.**

Response: We have now added a limitation to the paper.

Additionally, the absence of significant moderation by demographic variables may be due to several factors. For instance, limited variability or imbalance in demographic characteristics (e.g., more than 80% of the sample being Medicaid enrolled) within the sample, measurement limitations of screen-time categories, or potential model misspecification may have reduced the ability to detect interaction effects. It is also possible that these demographic factors do not meaningfully moderate the associations examined, suggesting that the relationships may be relatively stable across groups.

4. **In findings and discussion section, please clarify the interpretation of the relatively smaller differences in screen time observed on weekends and discuss whether uniformly high weekend screen use across groups may have attenuated between-group differences.**

Response: It is a great point. We have addressed it in the revised manuscript by expanding the discussion to explain the smaller weekend screen time difference. Weekend days are less structured, which may reduce the influence of contextual factors (e.g., after-school routines, parental supervision) that typically differentiate screen-use behaviors on weekdays. Therefore, both groups may substantially increase their screen time on weekends, leading to greater behavioral similarity and consequently smaller between-group differences.

5. **In data availability statement, clarify what types of de-identified or aggregated data and/or analytic code may be available upon reasonable request.**

Response: This has now been clarified in the data availability statement:

Upon reasonable request, de-identified and aggregated data summarizing children’s screen time behaviors (e.g., total, weekday/weekend, and domain-specific screen time) and basic demographic characteristics (age, sex, race, SES) and household income category) may be made available.

6. **As part of public health implications, please clarify how the findings particularly the role of weekday passive screen time can inform targeted intervention strategies.**

Response: Thanks for the suggestion. We have now revised the paper by citing a relevant Healthy People 2030 objective related to screen time mitigation.

The objective of increasing the proportion of parents who follow AAP recommendations on limiting screen time for children aged 6 to 17 years is a high priority issue within the Healthy People 2030 objectives, indicating significant public health implications. This study echoes the need for future screen time mitigation efforts to involve parents. Weekday screen behaviors were more influenced by change in parental regulation, indicating that weekday passive screen time may represent a particularly effective target for home-based intervention. Therefore, we suggested parents to prompt their child with frequent breaks from prolonged passive screen time and replace them with physical activity both indoor and outdoor. Additionally, they should establish family rules, goals, and routines to limit excessive screen time. Our findings emphasize the importance of targeted, parent-driven strategies that focus specifically on weekday routines.

**Reviewer #3**: Overall comments:

This cross-sectional online survey study reports differences in standing vs active screentime in OW and HW children from a predominantly lower income population. The results show that OW children engaged in more screentime and less active media time and overall have more screentime than HW children. As reported, the results are not new, although the use of a novel questionnaire provides additional nuance to the relationship between screentime and weight status. The authors do a good job excluding potentially problematic data and correctly note study limitations. However, there was no attention to the time of year (summer vs. school) that parents completed the survey, which seems to be an important factor given the Structured Day Hypothesis that the study was based on. This and some additional considerations are below.

Response: Thank you for your insightful review of our paper. We have carefully addressed your comments and provide below our responses to your comments point by point.

Regarding the timing of survey completion, this is a great insight. Inspired by your comment, we further examined the time stamps of the completed surveys. We found that 99.72% of the responses (n= 737 out of 739) were received between October 30 and December 17, and between January 8 and March 2, indicating that the screen time behaviors reported in this study primarily occurred during academic semesters. A small portion (n= 2, 0.27%) of surveys were completed outside the regular semester from December 20 to January 5 (e.g., during winter break). For this reason, we did not further analyze the group differences in screen time by summer vs. school days.

**Introduction**:

1. **The introduction was well-written, clearly pointed out the gap in knowledge, and was theoretically based. However, I do have some suggestions about the title of the paper and use of the term “socioeconomically disadvantaged” – The paper is primarily about the association between weight status and movement behaviors in a population that is predominantly, but not entirely socioeconomically disadvantaged. In fact, 27.4% of the families are categorized as high income (above the median income). I think the title overemphasizes the demographic characteristics and is not informative enough about the weight status differences (which were the primary finding)**.

Response: Thank you for your insightful feedback. To better reflect the primary focus and key findings of the study, we have revised the title to emphasize the association between children’s screen time and weight status rather than the demographic characteristics by revising the title to “Variations in Children's Screen Time Behaviors by Weight Status among a Mostly Disadvantaged Population”.

**Methods:**

1. **What was the justification for the age range used in the study? What age range has the Movement Behavior Questionnaire been previously used in?**

Response: Our phase 1 project sampling goal was to recruit a representative sample of children (parent proxies) following a multi-step recruitment plan (considering urbanicity and area deprivation). Children aged 5 to 12 represent the entry and the final stages of elementary education before entering middle or junior high school. This transition comes with substantial changes in peer dynamics and curriculum structures. Children aged 5 to 12 live in a relatively homogenous school structures and daily schedules, which enhances comparability across participants of their screen time behaviors. Therefore, we set up the age range for students to be 5-12 years old, corresponding to K-5 or K-6^th^ grades (elementary school grades in the state).

MBQ-C is a recently developed instrument to measure young children’s (e.g., ≤5 years old) 24-hour movement behaviors (self-reported by parent proxies). We used the MBQ-C questions that had content validity for this age range (as determined by our team with expertise in developmental psychology, behavioral science, and kinesiology) to comprehensively measure screen time (engagement type, posture, timing). We added a statement in the Discussion noting the need for additional validation and reliability investigation of the MBQ-C among older children or adolescent populations, as their screen time types and patterns may differ by age. We did not find a similar screen-time instrument with our questions of interest that had been validated in our age range, thus we accept this as a limitation of the study. Nonetheless, we believe these are important data to inform the field on screen usage in children prior to their transition to adolescence.

2. **It’s unclear why the question about “looking at photos” was included as part of the interactive screentime as this seems like a passive behavior. Can the authors justify this?**

Response: “Looking at photos” is regarded as an example of interactive screen time within the MBQ-C. Our understanding is that although the behavior may appear passive, it is categorized as an interactive because it typically involves some degree of user interaction and engagement with the device (e.g., scrolling, viewing, responding to, and perhaps sharing the photos).

3. **Lines 130-135 – how were parents provided with instructions or definitions for each of these categories?**

Response: The survey provided parent proxies with detailed descriptions of each screen-time behavior, including examples presented in the item header. The following questions asked the duration of the child’s engagement in that behavior across specific contexts. For example, a weekend screen-time item was introduced with the prompt “Thinking about the past week, on a TYPICAL WEEKEND DAY, how much time did your child spend playing games, looking at photos, or video chatting (e.g., FaceTime, Zoom, Skype) on a screen-based device such as a computer or laptop, video game console, iPad, tablet, or smartphone?” Subsequent items then asked respondents to report the total time spent engaging in the activity, as well as the amount of time spent standing during screen use.

4. **How was the sample size determined? Was any power analysis performed?**

Response: Power calculation was not conducted for this descriptive study. Our sampling goal was to recruit a large representative sample by age/grade, sex, area deprivation level, and urbanicity to identify disparities. The sampling process followed rigorous, multi-stage recruitment procedures as described in the Methods.

While no target sample size was predetermined, data collection continued until recruitment opportunities were exhausted, resulting in a final sample of 739 participants (filtered from 7373 responses). This sample size is comparable to or larger than those used in prior population-based studies of children’s screen time and was deemed adequate for estimating group differences.

5. **It is unclear from the sampling procedures if the time of year the survey was completed was corrected for in statistical analysis, or if only school time assessments were analyzed. According to the Structured Day Hypothesis, children’s screentime would vary depending on the time of year.**

Response: Thank you for raising this important point. As we explained earlier, 99.72% of the survey responses were collected during non-break time periods (n= 737 out of 739). Therefore, further seasonal adjustments were not made during the primary analyses.

**Results:**

1. **The authors report that ~25% of children met daily screentime requirements, but it’s unclear what recommendations were used for assessing daily screentime. Can this be added? Can the authors cite the specific guidelines that were used here?**

Response: The revised manuscript has clarified what the specific guideline refers to. We added the “2 hours or less per day” recommendation for recreational sedentary screen time. The corresponding guideline cited is at references [11–13].

2. **Line 202 – the authors report on the number of minutes children were engaged in media in the “sedentary posture” however, they did not actually assess children’s physical activity while they were watching media. The assumption is that children were sedentary while engaged in certain types of media, but we cannot verify this assumption. For this reason, the language on how media engagement is described should be tempered accordingly. The same can also be said about “standing” screentime. As activity was not objectively assessed, it’s unclear how parents reported minutes activities that were primary standing vs sedentary.**

Response: Yes, we have revised the language to avoid confusion. We have clarified that these are parent-reported child screen time while seated or standing as measured by the MBQ-C questions. We also have added a statement in the Limitations section acknowledging that possible misclassification of posture-related screen time categories may occur due to parent report.

3. **The authors reported no associations between demographic characteristics and movement / screentime, but I’m wondering if they looked at potential interactions between sex and age? For example, do weekend / weekday differences in weight related differences become more robust as children get older and drop out of sporting activities? Is this relationship exacerbated in girls relative to boys? These additional analyses may off the paper additional insight beyond what is largely already known (i.e., OW/OB demonstrate higher sedentary time and greater screen time).**

Response: We did perform two-way (and three-way) interaction analyses to explore the potential interactions between sex and age (and other factors) in understanding the potentially complex developmental patterns of screen time behaviors. However, all two-way (and three-way) interaction effects for screen time by demographic characteristics (age, sex, race, and SES) and weight status were non-significant in our analyses.

Discussion:

1. **There is no attention in the discussion to the potential effects that involvement in team sports and its associations with sex, age, and SES might have on the reported results.**

Response: We agree that participation in (team) sports could be one of the factors that would interact with demographic characteristics such as sex, age, and socioeconomic status and influence children’s screen time behaviors. However, we did not examine and measure sports participation in the study. To address this comment, we have added a statement to suggest future research looking into this interaction.
